# Traveling Letters, No. II

**Published:** 1844-08

**Authors:** Daniel Drake

**Affiliations:** New Orleans


### THE WESTERN JOURNAL.

New Series.—Vol. II.—No. II.

LOUISVILLE, AUGUST 1, 1844.

### TRAVELING LETTERS FROM THE SENIOR EDITOR.

NO. II.

New Orleans, May, 1844.

*To my Colleagues:*

Getlemen:—After writing to you from Mobile I returned to New Orleans, where I have spent the last two or three weeks, each of which has presented me with *materiel* for a long and desultory epistle, but the sedative influences of the South will protect both you and our readers from more than one.

I need scarcely tell you, that this place presents a sort of geological and social similitude. The mass of muddy, moving, waters which once mingled with the sea at this spot, brought down and deposited here particles and fragments of all the rocks, and soils, and forests of the heart of our continent. The same waters now bring down upon their turbid bosom the people and productions of the same lands. Hence the strata are not more compounded than the population which treads upon them. But here the analogy stops, for the strata are at rest, while the people are as agitated and restless as the atoms which float in the currents of the giant river. Such a spot presents much to interest and instruct the inquisitive physiologist and physician, and in commencing my letter, I scarcely know how to settle upon a particular topic. The hospitals of the city would seem, however, most appropriate for a medical traveler, and I shall therefore devote my letter to them, and the disease which supplies them with more patients than any other. Of these hospitals three are private and one public. Concerning the former, I shall say but little, as I wish to reserve ample space for the latter.

*Luzenberg's Hospital.*

This handsomely embellished and inviting establishment is situated hard by the Ponchartrain rail road, nearly two miles from the river, and consequently out of the city. The buildings are of wood, one and two stories high, and embosomed in trees and flowering shrubs, both native and exotic. By a contract with the general government, this is at present the hospital for sick seamen; it assumes, therefore, somewhat the character, as well as the appearance of a public institution.

*Maison de Sante.*

The owners, who are the physicians and surgeons of this hospital, are Drs. Stone, Kennedy and Carpenter. It stands in the edge of the city, on the corner of Canal and Claiborne streets, about three quarters of a mile from the river; is built of brick, plastered and penciled so as to resemble stone; has a verandah two stories high in the rear, and a lofty Doric portico in front. On one side of it there is a pleasant shrubbery, embracing many flowering exotics; and behind it, on Claiborne street, a supplemental house for colored patients.

*Circus Street Infirmary.*

This establishment, the property, and under the care of Drs. Campbell and Mackie, is situated in Circus street, near Poydras, and is nearer the steamboat landing than any other, and consequently more convenient to those who arrive from the upper country, ill with acute diseases. The buildings are of wood, well screened from the sun by verandahs, but destitute of surrounding trees and gardens.

These three private hospitals are at once creditable to their proprietors, and eminently useful to strangers, who cannot fail to be better nursed and looked after in either of them than in the hotels of the city. The physicians and surgeons, moreover, by whom they have been erected and are attended, are among the most scientific and experienced of the city; finally, the expense of a fit of sickness in one of them, will, in general, be less than in one of the hotels. I would, therefore, strongly recommend to our up-country friends, when taken ill in this 'city of strangers,' to make an early choice of one of these asylums. I say *early* for they need some prompting on this point. The diseases, especially the fevers of this place, are more rapid, and on the whole, perhaps, less painful than those of the interior, and are, therefore, very often allowed to advance to an incurable stage before they receive attention. To be successfully treated, they must be yielded to, and met with medicine in their first or forming stages, when, in general, they are quite as curable as those on the banks of the Ohio or Missouri.

Before proceeding to the great public infirmary of the city, I must speak a word or two concerning the unfinished

*Commercial Hospital.*

It was begun by the general government in or about the year 1836, and is roofed in. It is situated on the opposite side of the river, adjoining the open village of McDonoughville, and the site is well chosen, as it is more convenient to the ships and boats of the harbor, than it could have been placed on the city side. In company with Drs. Fearn, Carpenter, and Fenner, the last of whom is one of the editors of a forth-coming medical journal in this city, I made a visit to it a couple of days since. As a skeleton we were pleased with its appearance, and when its flesh and organs and skin shall be added, its appearance, as a public edifice, will not be unworthy of the nation, nor uninviting to the annual hundreds of salt and fresh water men who are sick at this vast commercial emporium. Why that which was begun eight years ago, and ought to have been erected eight years before, has been suffered to stop and retrograde in its development, is best known to those who are in the secrets of a government, which a political metempsychosis seems to animate with a new spirit every four years. The great river, on whose banks we stood while surveying this monument of culpable caprice, washes away the land in one place, and deposits land in another, to be in turn swept away; and thus it is with our much vaunted republican government; projects are begun and suffered to perish, *in embryo*, while some new scheme fixes the attention of one set of rulers, to be abandoned to premature decay by another. We would have ascended to the floorless portico of the edifice, but when half way up the unfinished stair case of Quincey granite, the tall weeds from beneath would but treacherously have supported our feet. In the rear, from under a projected verandah, we drove out the cattle which were browsing on the weeds and grass, and on looking into one of the future kitchens, we saw a dead cow lying on the ground in its centre. In the long cycle of changes in our administration, the Commercial Hospital of New Orleans, in which the whole Union is interested, may again take its turn.

### *The Charity Hospital.*

I come now to that public charity which is at once an honor to the city and State to which it belongs, and a blessing to thousands of the people of the United States and of Europe.

From Martin's History of Louisiana it appears, that a hospital was founded by Jesuits and Ursuline nuns, in the year 1727. It was under the care of the latter, and especially designed for the military. It was placed near the nunnery, at the corner of Chartres and Bienville streets. Subsequently, according to the *Historical Epitome of the State of Louisiana*, p. 323, there was a wooden hospital building for indigent persons, on Rampart street, between Toulouse and St. Peter streets, which was blown down in 1779.

An account of what followed for the next 30 years is condensed into a note to *Bullard and Curry's Digest of the Laws of Louisiana*, and is as follows:

"The Charity Hospital was founded by private donations until 1786, when a new building was erected by Dr. Roxas, and completed in 1786, at a cost of 114,000 dollars, and was called the 'New Charity Hospital of St. Charles.' The munificent founder furthermore endowed it with an annual revenue of 1,500 dollars, from the rents of stores, at the corner of St. Peter and Levee streets. It continued under the patronage and direction of the family of its founder and beneficent donor, until the 20th of February, 1811, when by an act of the Legislature of the Territory, it was made the duty of the Corporation of New Orleans to compromise with the patron of the Hospital of Charity, and establish it on a plan more perfect."

In 1814 an act was passed creating a Council of Administration, of which the Governor of the State is *ex-officio* the president, to manage all its affairs; since which a Vice President has been created. Two of the administrators visit the institution at least twice a week. By this act, every public ball and concert was to pay 10 dollars into the treasury of the hospital; and every theatre to give it four benefits a year. By the same act, the Governor was instructed to write to the Governors of the several States bordering on the Mississippi and Ohio rivers, soliciting subscriptions for the enlargement of the building. To this call the State of Pennsylvania nobly responded in 1817, by appropriating 10,000 dollars, but the other States remained silent. In 1818, the Legislature directed the sale of the edifice on Canal street, the purchase of a new site, and the erection of a more extensive and commodious building; which was accordingly done, at a cost of about 150,000 dollars; and the patients were transferred to it in the winter of 1833–'4. In 1837, the Legislature ordered, that the net proceeds, received into the State treasury, from all forféited bonds and recognisances, and all fines in criminal cases, and for contempt of court, should go to the hospital; provided, that more than 40,000 dollars should not be paid in one year. Notwithstanding this, it became necessary, in the very next year, to authorize the issue of State bonds to the amount of 100,000 dollars for the relief of the institution. By the same law, the four benefits a year from each theatre was commuted for 500 dollars per annum; each circus was taxed 100 dollars, and each show 50 dollars. In 1840, 40,000 dollars were appropriated out of the State treasury, of which 15,000 were to be applied to general objects, and 25,000 dollars to the erection of a lunatic asylum, for the State, on the same square with the hospital. Subsequently, another law, levying a tax on all passengers arriving in the city promised to render the revenues of the establishment sufficiently ample; but after a year its constitutionality was contested, and it is at present unexecuted. Such is a historical outline of the efforts of the State, for establishing and adequately endowing this noble public charity, as I have collected them from the Digest of

Laws quoted above, and from the printed reports of the administrators.

For a notice of the edifice itself I will depend on the 'Historical Epitome,' the general correctness of which I can avouch.

After mentioning that it stands on the square, bounded by Gironde, Gravier, St. Mary, and Common streets, this work says: "The New Charity Hospital, so called, is a building of great size, being about 290 feet in its total length, and three stories high. It is composed of a *corps de loges* in the centre, 116 feet front by 76 in depth, connected by the main building to the two wings, all three projecting in front and rear from the main edifice. The principal entrance, from Common street, is under a Doric portico, which covers the lower story of the *corps de loges*, into a spacious hall, intersected at right angles by another, running lengthwise the building on which the wards open. From this hall, access is had by broad stairs to the upper stories, which are similarly divided, and thus to the cupola, from which there is a magnificent view of the city and its environs." Within the building there are accommodations for the house physician and six students of medicine who wait upon the sick; an apothecary and his shop; and 18 or twenty sisters of charity. There are also two chapels, one for protestant, and one for Roman catholic worship. The edifice is designed to accommodate 540 patients, but during epidemics, a much larger number have been admitted.

I must now come to the inmates of this capacious establishment, the average annual number of which, for the last 14 years, has been 4,576, or 64,034 in all, as is shown by the tables published by the Administrators.

Of the great aggregate, but 625 were natives of Louisiana; many more were citizens of New Orleans by naturalization. Formerly the registers of the hospital did not distinguish between recent and old emigrants, but lately that has been done, and the two last annual publications of the Board of Administration, distinguish those patients who have been in New Orleans less than three years, from those who had been longer there. Nevertheless, this is not very exact, as the distinction relates to natives of Louisiana, as well as other States and foreign countries; but the number of persons born in that State who migrate to the city is not great. In 1842 and 1843, the number of patients admitted into the hospital was 9,421, of which 7,348 had been in the city less than three years. If we apply this ratio of about 78 per cent., to the number for 14 years—64,034—we have 49,944 persons resident less than three years in New Orleans, relieved at the hospital within that time. Of the foreigners, 50 per cent. have been Irish, nearly 14 per cent. German, 10 per cent. English, and something less than 8 per cent. French. The remaining 18 per cent., from various countries. New Orleans is at once the paradise and the grave of the half civilized Irish. Warm-hearted, gregarious, social, sensual, and reckless—fond of his shovel and his bottle, and cast upon a spot where the former is in demand and the latter within his reach, the generous-spirited Hibernian, on the mud-banks of the Mississippi, soon forgets the green hills of his native Shannon, and revelling for a brief space of time amid luxuries more abundant than he had ever dreamed of, is sent to the Charity hospital, whence he is transferred to fertilize the soil which but a few days before he might have been gaily up-heaving. But I must return from this rather inappropriate flight of fancy to our statistical tables.

Of the natives of the United States admitted, during 14 years, the following were from States lying in whole or in part within the Valley of the Mississippi:

| | | | |
|---|---|---|---|
| Pennsylvania | 3,281. | Tennessee | 614 |
| Virginia | 1,541. | Indiana | 284 |
| Kentucky | 943. | Missouri | 185 |
| Ohio | 910. | Illinois | 126 |
| Louisiana | 625. | Mississippi | 118 |
| | | Arkansas | 17——8,644. |

Of this number, less than twelve and a half per cent. are natives of Louisiana. Now, I will ask, is it either just or generous, that the support of a hospital, so extensively occupied by the citizens of other States, should devolve entirely on Louisiana, especially when but few of her citizens are found in the infirmaries of the States which crowd the wards of her own? It may be replied, that her commercial emporium is built up by the voyages which are made to it from those States; but to this it may be answered, that it is those voyages which advance the prosperity of the people who make them, and that the commerce which they sustain is reciprocally beneficial. To my own mind, it is a manifest duty of the States interested in the trade to New Orleans, to make occasional or annual appropriations to the support of the Charity hospital, according to the average number of their citizens, respectively, admitted into it.

I have another fact which goes to show how small a proportion of the patients of the Charity are residents of the city. It is the great number of men compared with women. In 16 months, being the whole of 1841 and part of 1844, 4,743 patients were admitted, of which 4,050 were males, and only 693 females. This disparity could not happen in an indigenous population, and shows that New Orleans is sought out by a vast number of the men of the States which dip into the great valley.

On the average number of patients in the hospital I have not much to say. At the end of February, 1844, there were 348, of March 395, of April 326, of May 316, averaging 346, in the healthiest portion of the year. Of course the number is much greater in autumn.

I have not time to analyse the registers of the hospital so far as to give you a view of the diseases of its inmates, but will select Autumnal and Yellow Fevers, as of deepest interest.

*Autumnal Fever.*—In 17 years, from 1825 to 1843 inclusive, there were admitted with various kinds of autumnal fever, 13,300 patients, or 782 annually. Of these 654 died, making about the ratio of 491 in 10,000. In four years, ending with 1833, 2,724 were admitted, of whom 174 died, or 639 in 10,000. In the four years ending with 1844, 4,478 were admitted, of which 121 died, or only 270 in 10,000; a difference in the ratio of mortality between the two periods, of 368 in 10,000;—an improvement which beautifully corresponds with the change of treatment from drastic purging and profuse salivation, to mild aperients, cupping, and the sulphate of quinine.

*Yellow Fever.*—Of all the hospitals in the United States, the Charity presents the most ample opportunities for studying this much dreaded and peculiar malady. I say peculiar, because my inquiries have nearly convinced me, that it should not be confounded with autumnal fever. It conforms, however, to several of the laws of that disease, as will be seen by the following tabular view, compiled with considerable labor, from *all* the records of the hospital. It exhibits the rise, number of patients, and deaths, from 1818 to 1843 inclusive.

STATISTICAL TABLE OF YELLOW FEVER FOR 26 YEARS.

| Years. | Time of the first 12 cases. | No. of Admittances. | No. of Deaths. |
|---|---|---|---|
| A. D. 1818 | From August 30 to Sept. 8 | 48 | 30 |
| 1819 | July 12 to 28* | 141 | unknown. |
| 1820 | July 21 to Aug. 9 | 122 | unknown. |
| 1821 | None. | None. | unknown. |
| 1822 | Sept. 1 to 7 | 349 | unknown. |
| 1823 | Aug. 23 to Sept. 11 | 2 | unknown. |
| 1824 | Aug. 4 to 26 | 176 | unknown. |
| 1825 | June 23 to July 12 | 92 | 59 |
| 1826 | May 18 to July 11 | 23 | 5 |
| 1827 | July 19 to July 30† | 388 | 265 |
| 1828 | June 18 to July 25 | 305 | unknown. |
| 1829 | May 23 to June 18 | 452 | unknown. |
| 1830 | July 15 to July 29 | 416 | 158 |
| 1831 | June 9 to Sept. 13 | 3 | 00 |
| 1832 | Aug. 15—unknown | 36 | 22 |
| 1833 | July 12 to Aug. 7 | 887 | 449 |
| 1834 | Aug. 28 to Sept. 30 | 150‡ | unknown. |
| 1835 | Aug. 23 to Sept. 1 | 505 | 284 |
| 1836 | Aug. 24 to Oct. 4 | 7 | 5 |
| 1837 | July 24 to Aug. 6 | 1,194 | 613 |
| 1838 | Aug. 25 to Oct. 2 | 24 | unknown. |
| 1839 | July 23 to Aug. — | 1,086 | 452 |
| 1840 | July 25 to Aug. 5 | 2 | 1 |
| 1841 | July 27 to Aug. 12 | 1,113 | 591 |
| 1842 | July 30 to Aug. 23 | 410 | 214 |
| 1843 | July 5 to July 29 | 1,053 | 487 |

From this table we find, that the time of the appearance of yellow fever ranges from the 18th of May to the 1st of September—a period of 15 weeks, and that the frequency of its beginning in the different months is as follows:—July, August, June, May, and September—oftener in either of the two former, than all the three latter taken together.

The following table, comprehending only three years, shows the relative prevalence of the disease in the different months:

* One case reported May 7.

† Imperfect, 12 days omitted.

‡ Imperfect.

TABLE.

| Years. | July. | August. | Sept. | October. | Nov. | Dec. |
|---|---|---|---|---|---|---|
| 1841 | 0 | 177 | 642 | 252 | 37 | 8 |
| 1842 | 1 | 46 | 247 | 93 | 23 | 0 |
| 1843 | 23 | 188 | 365 | 351 | 111 | 15 |
| Average | 8 | 137 | 418 | 232 | 57 | 7⅔ |

Thus the order of predominance in these three years, is September, October, August, November, July, and December, the two latter, however, being nearly equal.

I must now give you some of the statistics of death. In 10 years lying between 1825 and 1843, 5,973 patients with yellow fever, were admitted, of which 2,967 died, or within a fraction of 50 per cent. We may place it at that. If we add to the number admitted in those years, the numbers for the years the mortality of which was not recorded, we have an aggregate of 8,758—one-half of whom we may assume died, giving an annual average mortality, for 26 years, a fraction more than 172.

The mortality of 50 per cent. may seem large, and is indeed large, compared with that in the private practice of our brethren in New Orleans. But it should be stated, that a great number of the patients are admitted in a moribund condition—are sent to the hospital to die, that they may be buried at the public expense. In fact, from the roaming and reckless character of those who depend on the hospital in sickness, we may presume that but few of them find their way to it in the early stage of the disease.

I have sought to ascertain whether the treatment of the malady is undergoing any amelioration; and will give you the comparison of two periods, each of four years. In 1825, '27, '30, and '33, 1,783 were admitted, of whom 931 died, giving a mortality of 52.21 per cent., or 5,221 in 10,000. In the years 1839, '41, '42 and '43, 3,662 were admitted, and the deaths were 1,747, equal to 47.70 per cent., or 4,770 in 10,000. The difference of 4.21 per cent., or 421 in 10,000 in favor of the last period, during which a milder treatment has been pursued, clearly indicates, either a less malignant disease, or a more appropriate method of cure—I shall assume the latter.

The question is often asked, does yellow fever occur in New Orleans every year? By reference to the statistical table before me, I find that of 26 years, 5 produced either none or less than 10; five produced more than 10 but less than 100; the other 16 more than 100. The second group, and the last year of the first, rather arbitrarily separated from it, deserve especial notice, as presenting the disease in a sporadic form, and, therefore, bearing on the question of local origin, or importation. It may be said, that the disease has been epidemic about two-thirds, and sporadic about one-fifth of the years, for the last 26, and, consequently, that it prevails almost as certainly in New Orleans, as does autumnal fever in any of our country towns, and far more frequently than measles or scarlatina invades either.

It has been said, that the disease is epidemic every other year, but the table to which I have referred shows this to be a fallacy; not that such has not been the case, but that it was decidedly prevalent from 1827 to 1830 inclusive; from 1833 to 1835; and from 1841 to 1843. On the other hand, no period of three years, and but one of two, has elapsed since 1818, in which it was only sporadic.

A few additional observations will bring this tedious epistle to a close.

Private patients are accommodated in the Charity. Poor people of the city sometimes resort to it in sickness, and strangers may be made very comfortable in it.

A part of its revenues, amounting to something like $1,500 a year, is derived from a charge of 25 cents at the gate to those who visit the institution. This is not exacted from the near relatives of the sick.

I am sorry to feel it my duty to reiterate, that the registers of the hospital have been very imperfectly kept. 1st. The names of the diseases, epidemic cholera excepted, for the first four months of 1834 are wanting. 2d. The record is lost from 18th of September to 13th of October, 1832. 3d. There is no uniformity in the names given to the same disease. 4th. The clerk at the gate, who admits the patient, is absurdly ordered to name his disease, a task impracticable, under the circumstances even for a physician, producing a record of no value, and at the same time calculated to mislead; for the attending physician not unfrequently neglects to make to the clerk any return of the malady, and at the end of the month when a report is made to the Administrators, the names given by the clerk, become permanent.

Without multiplying these specifications, I will observe that at the present time the medical gentlemen in attendance, appear wide awake to these and some other defects, and will no doubt do all that can be done to remedy them. There are, however, two difficulties in the way; first, the perpetual succession of new medical and surgical attendants; second, the want of meetings of consultation and co-operation.

The wards of the hospital are admirably ventilated and kept remarkably clean; indeed, the whole interior exhibits evidence of the superintending hand of woman. I was introduced to the Sister superior, appropriately called *Regina*, and found her dignified, intelligent, and well-informed on the affairs of the establishment. She and the sisterhood over which she presides, in connexion with the amiable and well-informed house physician, Dr. Waverstrandt, direct and see executed the entire internal economy of the establishment —the former receiving little other compensation than their support in the institution. Thus they are truly *Sisters of Charity*, and the sick stranger may receive his bitter dose from one of them as from the hand of the sister whom he has left behind him.

But I must close this communication to resume the inquiries which demand all my time, and which so press upon me, that I fear there are many inaccuracies in what I have so hastily sketched off.

Your very obedient servant,
DANIEL DRAKE.

---

NO. III.

New Orleans, May, 1844.

